# Fundamentals of a healthy and sustainable diet

**DOI:** 10.1186/s12937-024-01049-6

**Published:** 2024-11-30

**Authors:** Mark Lawrence

**Affiliations:** Institute for Physical Activity and Nutrition, School of Exercise and Nutrition Sciences, Deakin University, Geelong, VIC Australia

**Keywords:** Healthy and sustainable diet, Dietary guidelines, Dietary variety, Dietary balance, Dietary moderation, Ultra-processed foods, Food selection guide

## Abstract

**Background:**

A healthy and sustainable diet is a prerequisite for population and planetary health. The evidence of associations between dietary patterns and health outcomes has now been synthesised to inform more than 100 national dietary guidelines. Yet, people select foods, not whole dietary patterns, even in the context of following specific diets such as a Mediterranean diet, presenting challenges to researchers, policymakers and practitioners wanting to translate dietary guideline recommendations into food-level selection guidance for citizens. Understanding the fundamentals that underpin healthy and sustainable diets provides a scientific basis for helping navigate these challenges. This paper’s aim is to describe the fundamentals of a healthy and sustainable diet.

**Results:**

The scientific rationale underpinning what is a healthy and sustainable diet is universal. Everyone shares a physiological need for energy and adequate amounts, types and combinations of nutrients. People source their energy and nutrient needs from foods that are themselves sourced from food systems. The physiological need and food systems’ sustainability have been shaped through evolutionary and ecological processes, respectively. This physiological need can be met, and food systems’ sustainability protected, by following three interlinked dietary principles: (i) Variety – to help achieve a nutritionally adequate diet and help protect the biodiversity of food systems. (ii) Balance – to help reduce risk of diet-related non-communicable diseases and excessive use of finite environmental resources and production of greenhouse gas emissions. (iii) Moderation – to help achieve a healthy body weight and avoid wasting finite environmental resources used in providing food surplus to nutritional requirements.

**Conclusion:**

The fundamentals of a healthy and sustainable diet are grounded in evolutionary and ecological processes. They are represented by the dietary principles of variety, balance and moderation and can be applied to inform food-level selection guidance for citizens.

## Background

A healthy diet is a prerequisite for a healthy life. Everyone shares a physiological need for energy and adequate amounts, types and combinations of nutrients. These are needed for optimal growth, development, maintenance, and repair of the body and as fuel for an active and healthy life [1]. Conversely, when we are unable to consume a healthy diet, we may be at risk of malnutrition in all its forms which include undernutrition; micronutrient deficiencies; and overweight and obesity and resulting in diet-related noncommunicable diseases (NCDs), such as cardiovascular disease and diabetes [2]. From these human health and well-being perspectives, a healthy diet has been defined as one that “promotes optimal human growth and development and prevents malnutrition in all its forms” [1].

Historically, understandings of what constitutes a healthy diet were framed in terms of the consumption of adequate amounts of nutrients to prevent nutrient deficiency diseases [3]. As nutrition science has evolved we have learned more about associations between dietary patterns, i.e. “the quantities, proportions, variety, or combination of different foods, drinks, and nutrients in diets, and the frequency with which they are habitually consumed” [4], and health outcomes. The evidence for these associations has now been synthesised to inform more than 100 national dietary guidelines [5]. Also, there has emerged an increasing amount of evidence showing that what we eat can impact environmental sustainability [6] and in turn, how environmental disruptions can impact food production systems [7]. Recognition that there are co-benefits for population and planetary health from adopting diets that are both healthy and sustainable has led to an environmental sustainability dimension being incorporated into many national dietary guidelines [8–10].

Because people usually select foods, rather than whole dietary patterns, even in the context of following specific diets such as a Mediterranean diet, food policymakers and practitioners seeking to promote healthy and sustainable diets face an intractable challenge of needing to translate dietary guideline recommendations into more accessible food-level advice for citizens. This challenge leads to practical scientific uncertainties with assessing a food’s ‘healthiness’ [11, 12] and sustainability [13]. This paper’s premise is that an understanding of the fundamentals that underpin healthy and sustainable diets provides a scientific basis for helping navigate these uncertainties and informing food selection guidance.

The aim of this paper is to describe the fundamentals of a healthy and sustainable diet. Here, a healthy and sustainable diet is defined as one that promotes optimal human growth and development and prevents malnutrition in all its forms, as well as having low environmental impact to protect food and nutrition security for present and future generations. The paper addresses the aim in three stages. First, it describes the scientific rationale underpinning the links between dietary patterns and health and environmental sustainability outcomes. Second, it synthesises this science into principles for selecting a healthy and sustainable diet. Third, it translates those principles to food selection practice to achieve healthy and sustainable diets. The information presented in this paper relates to the fundamentals of a healthy and sustainable diet for healthy free-living children and adults, not for treating specific health conditions or food safety issues. The ability to select a healthy and sustainable diet based on these fundamentals is dependent on the social, political, environmental and economic determinants of a food system’s structure and operation which influence how and why food is supplied and dietary choices are made [14].

## Scientific rationale underpinning the links between dietary patterns and health and environmental sustainability outcomes

For millions of years, dietary patterns were determined by the availability of a wide range of minimally processed, ‘nutritious’ foods, i.e., foods which are sources of energy and nutrients. Requirements for energy and nutrients were shaped by evolutionary processes as human physiology continually adapted to shifts in dietary patterns [15]. The food systems from which foods were sourced existed in harmony with ecosystems operating within ‘planetary boundaries’ [16]. Progressively, food processing innovations were introduced to help protect food safety, reduce spoilage of highly perishable foods, make food more edible at the household level and increase food appeal among citizens.

Modern dietary patterns have transitioned significantly from those present during most of human history [17]. This transition is a consequence of rapid urbanization and changing lifestyles intertwined with food supply changes characterised particularly by the widespread displacement of minimally processed nutritious foods for ‘ultra-processed foods’ (UPFs). Ultra-processed foods are formulations of ingredients, mostly of exclusive industrial use, typically created by a series of industrial techniques and processes [18]. Human physiology has not been able to adapt sufficiently to modern dietary pattern exposures, contributing to diet being a leading risk factor for the global burden of disease [19]. Concurrently, ecosystems have not been able to adapt sufficiently to the operations of modern food systems that are fostering these modern dietary patterns, resulting in food systems being a leading contributor to multiple planetary boundary transgressions [6].

## Synthesising the science into principles for selecting a healthy and sustainable diet

There are three principles that capture the scientific rationale when determining healthy and sustainable diet recommendations, they are dietary: variety; balance; and moderation. The principles need to be considered as a coherent whole with all three being equally important and interlinked. A description of each principle and how they relate to a healthy and sustainable diet follows.

### Principle 1: Dietary variety – to help achieve a nutritionally adequate diet and help protect the biodiversity of the food supply

Dietary variety refers to eating a variety of nutritious foods every day. Apart from breast milk in the first six months of life, no single food can supply an appropriate balance of all the nutrients necessary for health. Foods with similar characteristic nutrients and nutrient profiles can be grouped into distinct food groups based on their type and nutrient composition [5]. Many countries with food-based dietary guidelines identify 4–6 ‘core’ food groups: starchy staples, vegetables, fruits, dairy foods, other ‘protein foods’ and fats and oils [5]. A nutritionally adequate diet can best be achieved by selecting foods from both across and within the core food groups while prioritising minimally processed foods. These foods should be consumed in amounts recommended in dietary guidelines. A number of countries have undertaken dietary modelling activities to estimate a minimum number and reference size of food serves to be eaten daily from each food group to enable nutrient intake recommendations to be met [20–22].

From a health perspective, foods can differ substantially in their composition of nutrients and more than an estimated 26,000 other bioactive compounds [23]. Dietary variety increases the likelihood of consuming an adequate amount, type and combination of nutrients and other bioactive compounds for promoting nutritional health and preventing nutritional deficiency diseases. Synergistic effects among nutrients within and between foods are also instrumental in varied diets positively impacting on health [24]. Conversely, risk of consuming excessive amounts of certain nutrients and other bioactive compounds that may be present in high concentrations in some foods may be reduced through dietary variety.

From a sustainability perspective, consuming a variety of foods helps to protect the biodiversity of food systems by promoting the production of a wide range of genetically diverse food crops and species. Food crop and species biodiversity helps increase the resilience of food production to threats from pests and diseases in agroecosystems [25]. This biodiversity may also help contribute to higher and more stable yields as well as lower land clearing and use of harmful agrochemicals [26].

### Principle 2: Dietary balance – to help reduce risk of diet-related non-communicable diseases and excessive use of finite environmental resources and production of greenhouse gas emissions

Dietary balance refers to the relative dietary proportions of the different food groups from which foods are selected. Dietary imbalances arise when the total amount of foods from one or more food groups is consumed in excessive or inadequate amounts relative to the total amount of foods consumed from other food groups. Two dietary imbalances are receiving particular attention. First, in many high-income countries, it is reported there is too low an intake of nutritious plant-source foods relative to animal-source foods [27, 28]. Second, in an increasing number of countries around the world, it is reported there is too low an intake of minimally processed nutritious foods relative to UPFs [10, 27, 29–31].

From a health perspective, dietary imbalances are associated with an increased risk of diet-related NCDs such as obesity, cardiovascular disease, certain cancers and diabetes [32, 33].

Concerning the imbalance of nutritious plant-source foods relative to animal-source foods, the World Health Organization (WHO) has advised that a healthy diet includes [34]:

- Fruit, vegetables, legumes (e.g. lentils and beans), nuts and whole grains (e.g. unprocessed maize, millet, oats, wheat and brown rice).
- At least 400 g (i.e. five portions) of fruit and vegetables per day [33], excluding potatoes, sweet potatoes, cassava and other starchy roots.

Currently, the WHO (and other UN agencies) does not provide explicit advice on quantities of animal-source foods to include in a healthy and sustainable diet.

Concerning the imbalance of minimally processed nutritious foods relative to UPFs; UPFs are not an essential component of a healthy and sustainable diet. These foods often contribute superfluous energy, ‘risk’ nutrients (added sugar, salt and industrial trans fatty acids) and industrial ingredients, while displacing minimally processed nutritious foods, from dietary patterns [18]. The WHO has advised that a healthy diet includes [34]:

- Less than 10% of total energy intake from free sugars.
- Less than 30% of total energy intake from fats. Unsaturated fats are preferable to saturated fats and trans-fats of all kinds.
- Less than 5 g of salt per day. Salt should be iodized.

Reducing excessive dietary intake of UPFs will help correct dietary imbalances not only directly by reducing dietary intake of risk nutrients and industrial ingredients, but also indirectly by increasing dietary intake of minimally processed nutritious foods (assuming the maintenance of a relatively constant dietary energy intake). The amount of UPF that might be accommodated within a healthy diet will vary with a person’s nutrient and energy requirements.

From a sustainability perspective, consuming an excessive amount of animal-source foods relative to nutritious plant-source foods can have significant adverse impacts in terms of greenhouse gas emissions, land use and degradation, water use and pollution linked to nutrient-rich fertilisers, and other environmental indicators [35, 36]. Consuming an excessive amount of UPFs relative to minimally processed nutritious foods is associated with biodiversity loss and soil degradation, excessive use of finite environmental resources such as water and food packaging waste, especially plastics [37, 38]. These multiple adverse sustainability impacts resulting from excessive consumption of UPFs are even more concerning given these foods are surplus to nutritional requirements.

### Principle 3: Dietary moderation – to help achieve a healthy body weight and avoid wasting finite environmental resources used in providing food surplus to nutritional requirements

Dietary moderation refers to consuming enough food to provide for but not exceed the body’s energy needs. It is essential for optimal growth and development (until physical maturity is reached), to maintain a healthy body weight and composition (post-maturity), and to allow for a level of physical activity consistent with long-term good health (all ages) [32].

From a health perspective, people who consume enough food to provide for but not exceed their body’s energy needs generally have a healthy body weight and composition, and a lower risk of experiencing many diet-related NCDs, such as cardiovascular disease and type 2 diabetes, relative to people whose energy needs are exceeded. People who consume an excessive number and/or size of food serves from each food group beyond that necessary to meet their body’s energy needs may become overweight or obese. Living with overweight or obesity is associated with metabolic problems and an increased risk of many diet-related NCDs [39]. Conversely, children unable to consume enough food to meet their energy needs may have their growth and development stunted. Stunting is also associated with poor physical, mental and functional health problems [40]. Adults unable to consume enough food to meet their energy needs over an extended period will suffer from undernutrition and will be vulnerable to adverse health outcomes [41]. Both stunting and underweight are a consequence of challenges with food accessibility, affordability and availability [42].

From a sustainability perspective, consuming excessive amounts of food not only contributes to overweight and obesity, but also because it is surplus to meeting the nutritional needs of the consumer it is a waste of the finite environmental resources used in producing, processing, distributing, storing and preparing that food [43]. Also, excessive food consumption usually involves extra food being purchased and subsequently leading to extra amounts of food packaging requiring disposal.

## Translating the dietary principles to inform food-level selection guidance

In practice, determining the precise amounts, types and combinations of foods which constitute a healthy and sustainable diet will vary depending on individual characteristics such as age, gender, lifestage and lifestyle (e.g., work practices which may be sedentary or involve heavy manual labour), as well as cultural, social and environmental circumstances. However, the principles of a healthy and sustainable diet apply equally to individuals and populations around the world. Dietary guidelines have remained broadly consistent over several decades for most countries and they are remarkably similar across the different countries [44], indicating that the fundamentals of a healthy diet persist over time and place.

**Fig. 1:**
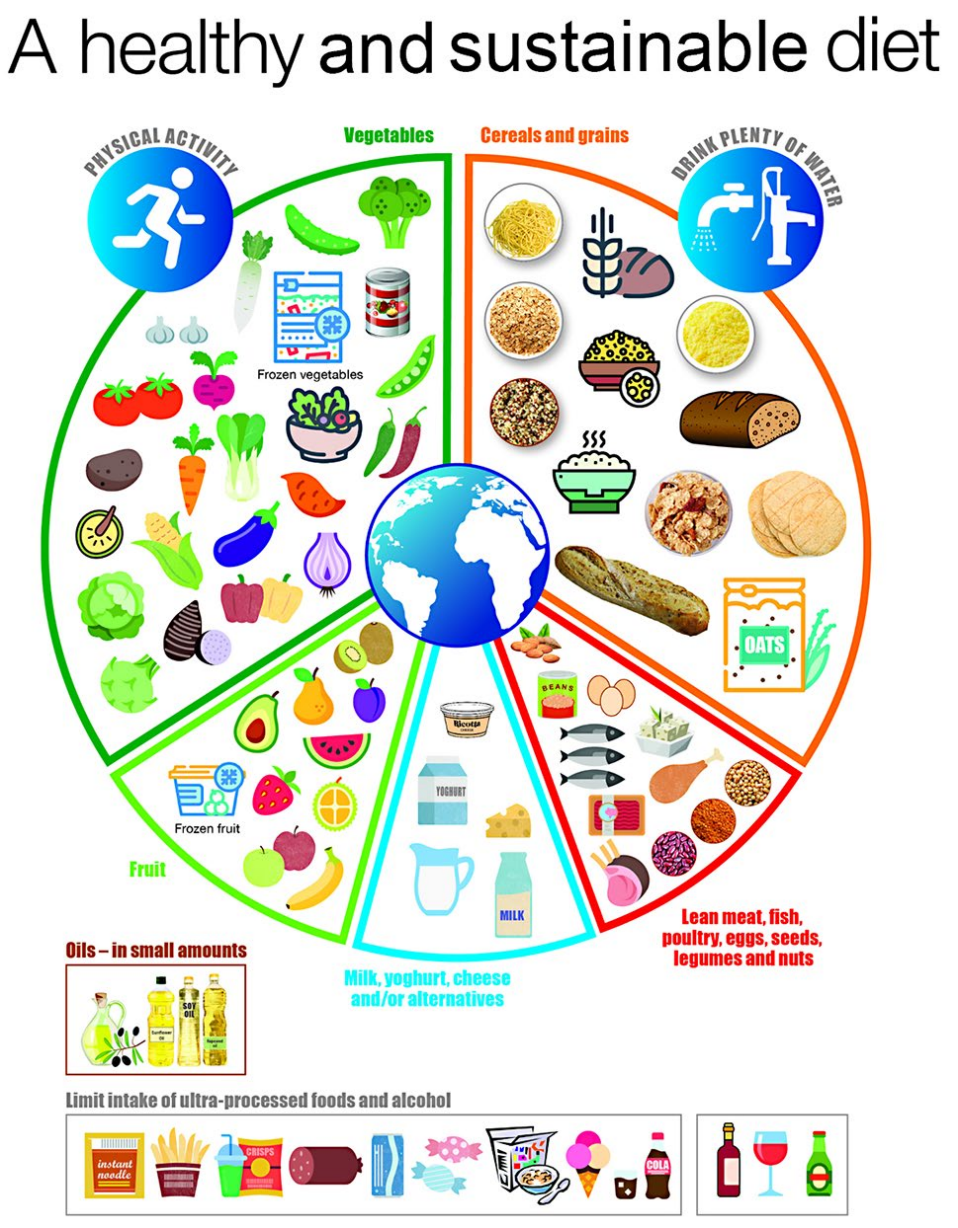
Generic healthy and sustainable food selection guide*. * ‘Designed by minimum graphics’. Copyright for this illustration belongs to the World Health Organization

Many countries and regions have published a food guide as a graphic representation or visualization of their dietary guidelines to show how the dietary principles of variety, balance and moderation apply to their food supplies, eating habits and social and cultural circumstances [30, 45, 46]. The visual features and certain technical details of these guides vary between countries. Nevertheless, the nutrition science principles that inform these guides are broadly similar. In practical terms these food guides help translate dietary guideline recommendations into food selection advice. Figure 1 presents a synthesis of the common features and technical criteria of national food guides compiled by the Food and Agriculture Organization [5]. This generic guide promotes the application of each of the dietary principles in the following ways:

### Variety

- *How the principle might be depicted*: Nutritious foods with similar characteristic nutrients and nutrient profiles are grouped together into 5 core food groups (Vegetables; Fruit; Cereals and grains; Lean meat, fish, poultry, eggs, seeds, legumes and nuts; and Milk, yoghurt, cheese and/or alternatives).
- *How the principle might be described in accompanying text*: ‘Each day choose a variety of foods from within each of, and across all, the 5 core food groups.’

### Balance

- *How the principle might be depicted*: In relation to the balance of nutritious plant-source foods to animal-source foods - the relative size of each core food group segment is broadly indicative of the recommended number of food servings from that group e.g., food groups containing predominantly nutritious plant-source foods will make up a larger proportion of the guide than food groups containing predominantly animal-source foods. In relation to the balance of UPFs to minimally processed, nutritious foods - an ‘Ultra-processed foods’ food group is separated from the 5 core food groups. Defining this non-core food group in terms of the UPF concept provides a stronger conceptual (grounded in evolutionary and ecological processes) and evidential basis than the mostly indeterminate current descriptors such as ‘Extras’ or ‘Discretionary’.
- *How the principle might be described in accompanying text*: ‘Each day choose foods from all 5 core food groups in overall amounts equivalent to the food group proportions set out on the guide’, and ‘Limit intake of UPFs’.

### Moderation

- *How the principle might be depicted*: Illustrations show moderate portion sizes for foods from each food group.
- *How the principle might be described in accompanying text*: ‘Enjoy eating when you’re hungry though stop when you feel full, avoid eating an excessive number and/or size of food serves from each food group and eating when you don’t have a sensation of hunger’(adapted from [47]).’

The generic food guide is intended to serve as an example and therefore, be flexible with a diversity of foods from around the world illustrated in each of the food groups so that the healthy and sustainable diet principles can be adapted to different food habits, food supplies, eating habits and social and cultural circumstances in each country context.

The food guide illustrates four other considerations for healthy and sustainable diets:

i) Safe drinking water, preferably tap or portable water or water from other improved sources (e.g., protected boreholes) in preference to other drinks, especially sugar-sweetened beverages [48] and bottled water, is an integral component of a healthy and sustainable diet.
ii) Food and drinks to limit are predominantly UPFs and alcohol which are nutritionally poor and not an essential component of a healthy diet.
iii) Use small amounts of unsaturated vegetable oil (e.g. olive, soy, sunflower, corn or rapeseed) when required, though many Mediterranean countries report that olive oil makes an important contribution to the health benefits of Mediterranean diets and recommend it be generously included in food preparation and dressings for such diets [49–51].
iv) Physical activity is an important complement to a healthy diet.

## Concluding comments

This manuscript has described how the fundamentals of healthy and sustainable diets are grounded in evolutionary and ecological processes. It reports how the science identifying dietary associations with population and planetary health outcomes can be synthesized into three dietary principles and translated into food-level selection guidance. These principes are commonly represented in national dietary guidelines and food selection guides, albeit with adaptation for cultural, social and environmental circumstances.

The unprecedented rate and scale of change in food systems and environmental settings will continue to outpace the ability of human physiology and ecosystems to adapt and evolve. The bidirectional relationship between dietary patterns and the ecosystems may present an increasing number of trade-offs to consider when preparing dietary guidance for promoting healthy and sustainable diets. For example, there are complex environmental and health considerations when comparing diets containing high amounts of animal-based foods such as meat, chicken and eggs, plant-based foods such as legumes and vegetables which are minimally processed, and novel plant-based food products designed to mimic and replace animal-based foods and which generally are ultra-processed. Plant- and novel plant-based diets typically have smaller environmental footprints than animal-based diets though the alignment of their nutrient profiles with healthy diets is mixed and those containing high amounts of ultra-processed products are associated with longer term adverse health outcomes [52]. One study modelling dietary recommendations for reducing the amount of animal-source foods in diets, in a large part to mitigate greenhouse gas emissions, showed that dietary intakes of vitamin B12, calcium, iron, and zinc would be below the recommended nutrient intakes for adults and women of reproductive age [53]. These dynamic circumstances highlight the importance of policymakers being able to discern the level of processing of different types of plant- and novel plant-based foods as well as using nuanced decision-making approaches to consider the range of environmental and health benefits and risks of these foods. A future priority will be constant investigation of associations between dietary patterns and health and sustainability outcomes as well as ongoing review of the evidence base that informs dietary guidelines.

Into the future, guidance on healthy and sustainable diets will need to continue to be based on the synthesis of the best available evidence of associations between dietary patterns and physiological, and increasingly planetary, health outcomes. Yet, dietary guidelines do not speak for themselves. The investment in their preparation will need to extend to investment in their promulgation to nutrition researchers, policymakers and practitioners, and their subsequent translation into food-level selection guidance for citizens.

## Data Availability

No datasets were generated or analysed during the current study.

## Abbreviations

NCDs: Noncommunicable diseases
UPFs: Ultra-processed foods
WHO: World Health Organization
